# Supplementary figures and images for: Fn14 promotes myoblast fusion during regenerative myogenesis
Source: Life Sci Alliance. 2023 Oct 9;6(12):e202302312. doi: 10.26508/lsa.202302312 (PMC10561765; doi:10.26508/lsa.202302312)

**FIGURE 1B.**

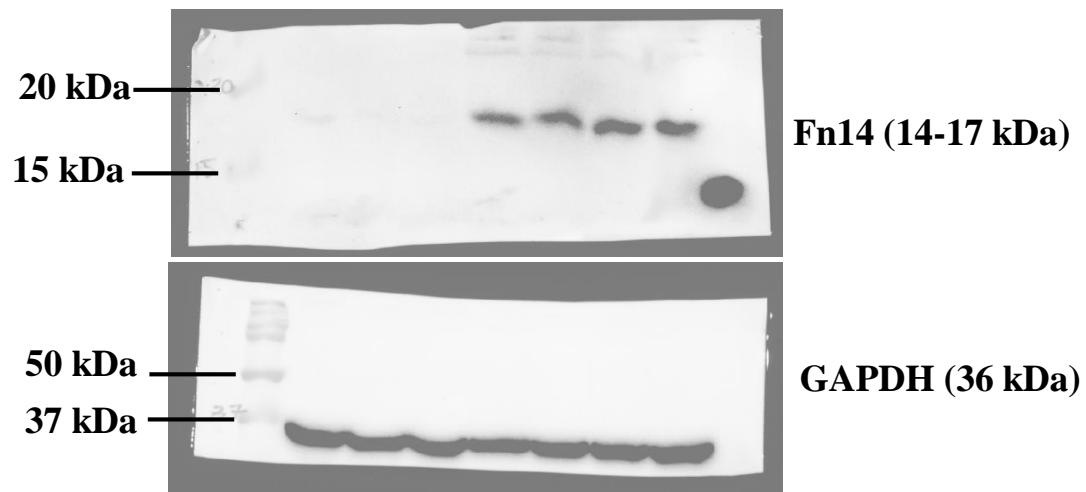

**FIGURE 1I**

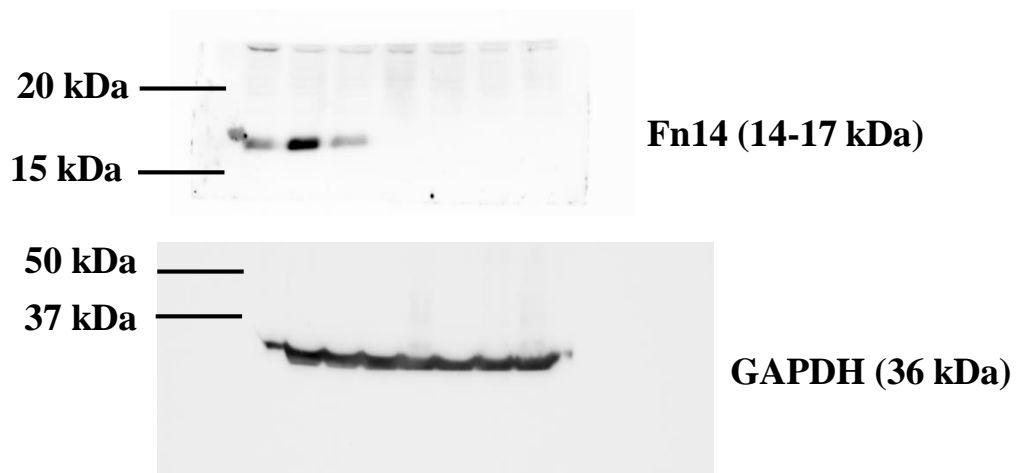

Supplement: Supplementary file 1 [file LSA-2023-02312_SdataF1.pdf]

**Supplemental FIGURE S1C**

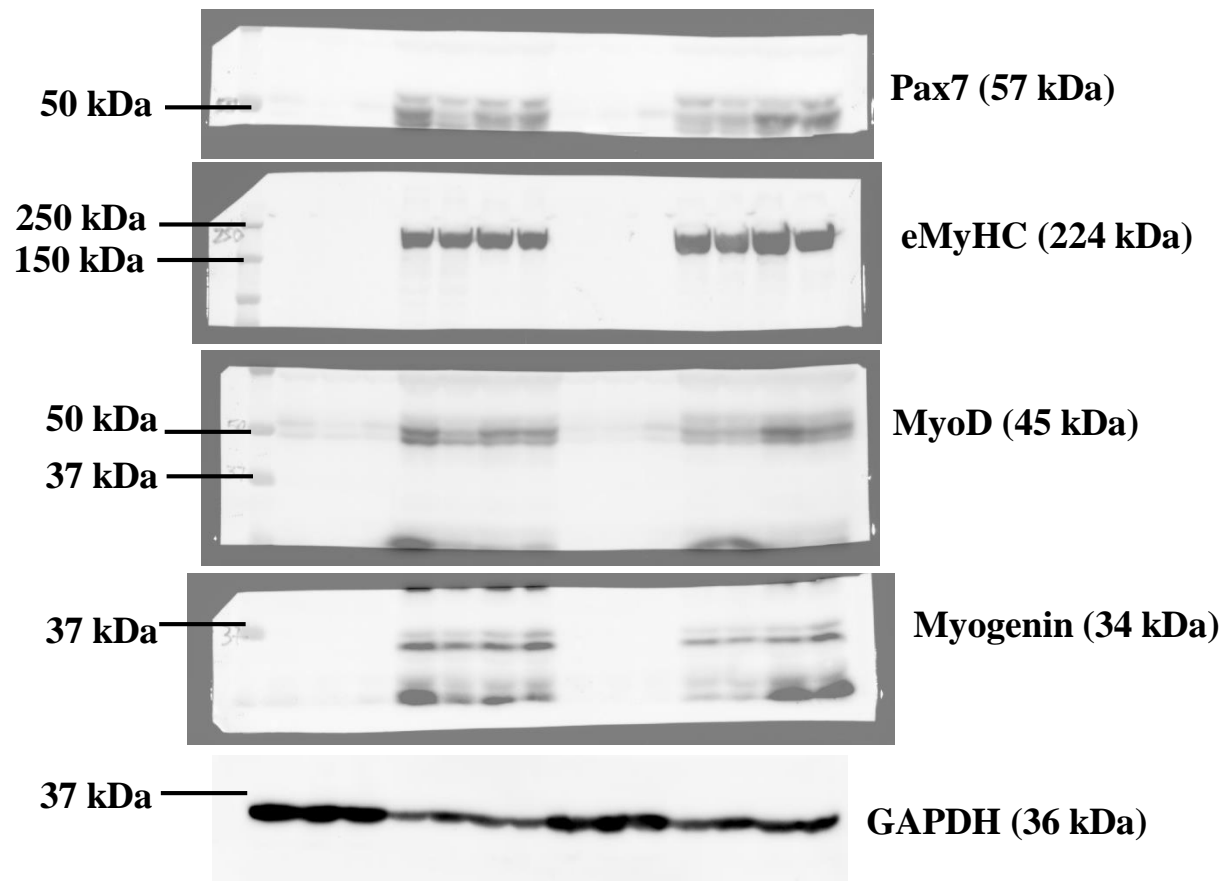

Supplement: Supplementary file 2 [file LSA-2023-02312_SdataFS1.pdf]

**FIGURE 3B.**

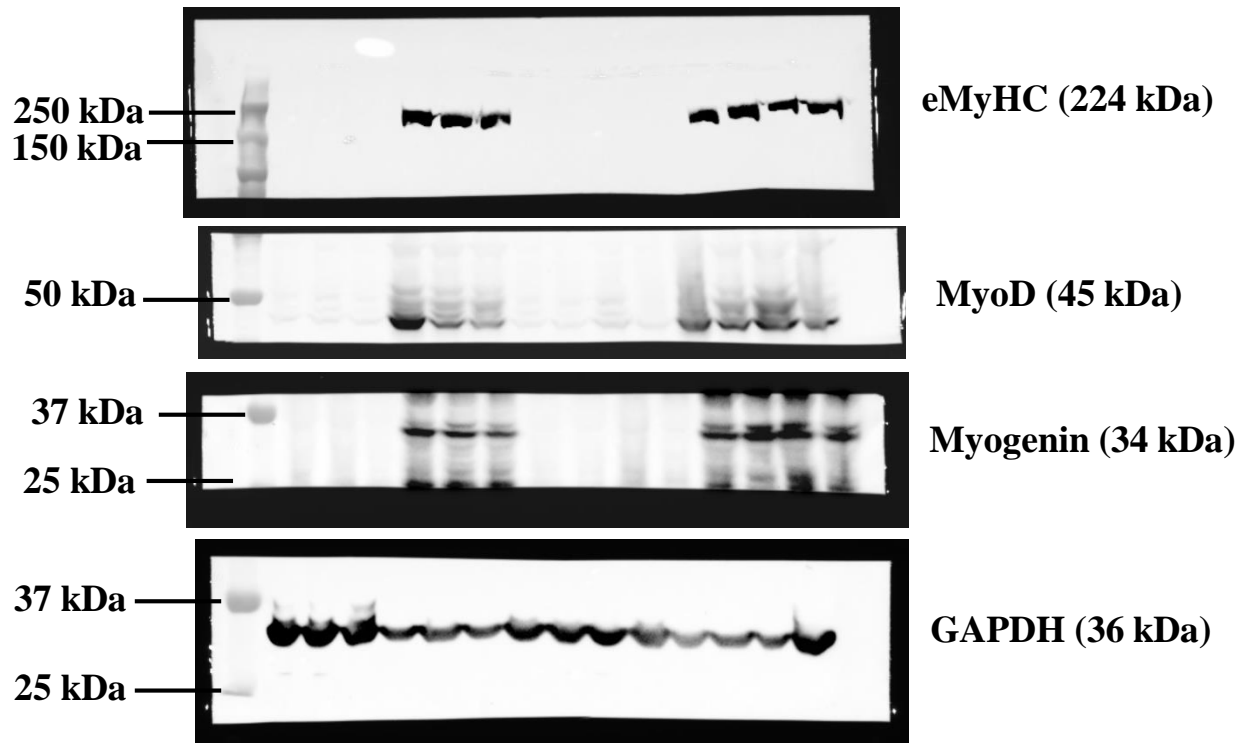

Supplement: Supplementary file 3 [file LSA-2023-02312_SdataF3.pdf]

**FIGURE 5G.**

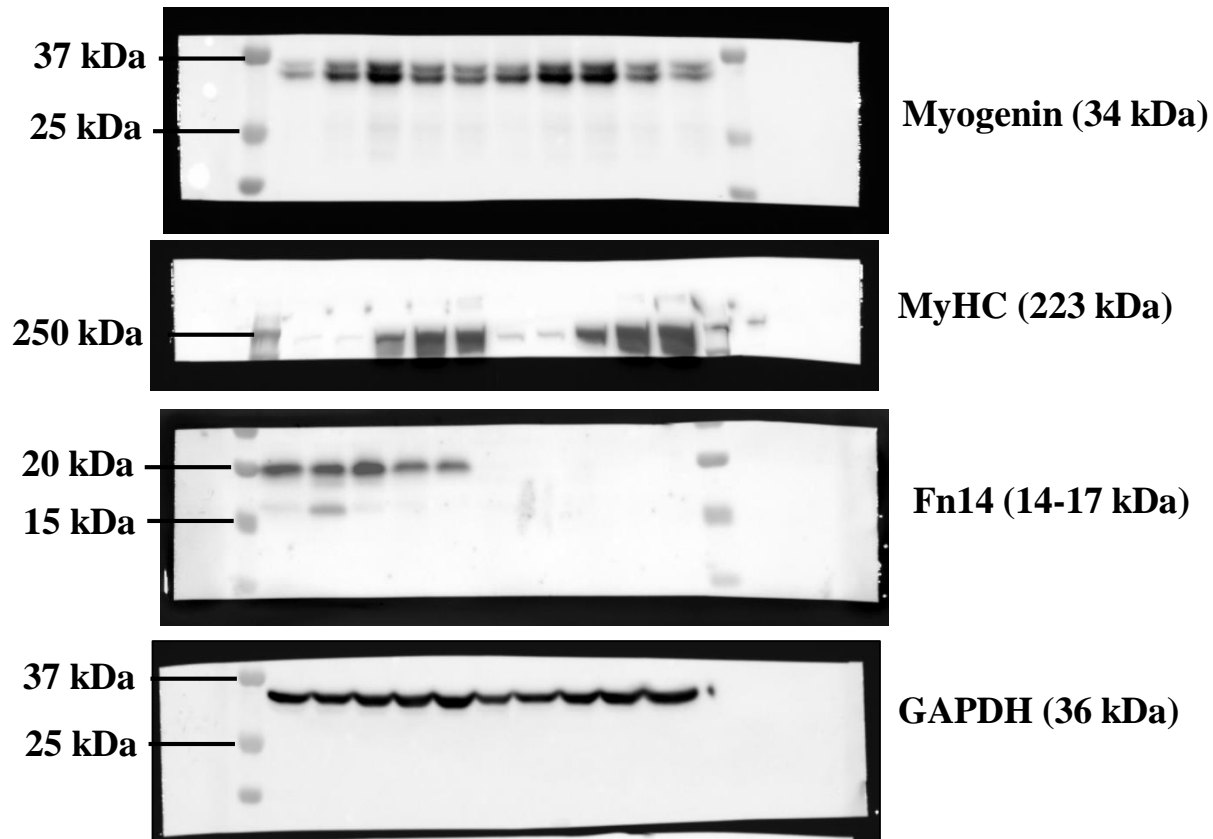

Supplement: Supplementary file 4 [file LSA-2023-02312_SdataF5.pdf]

**Supplemental FIGURE S6A**

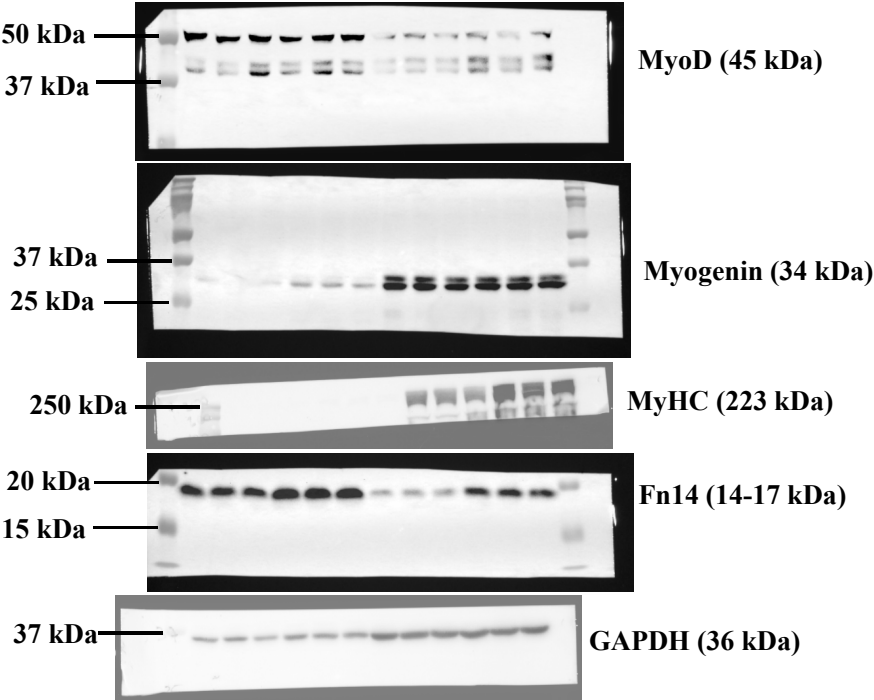

**Supplemental FIGURE S6C**

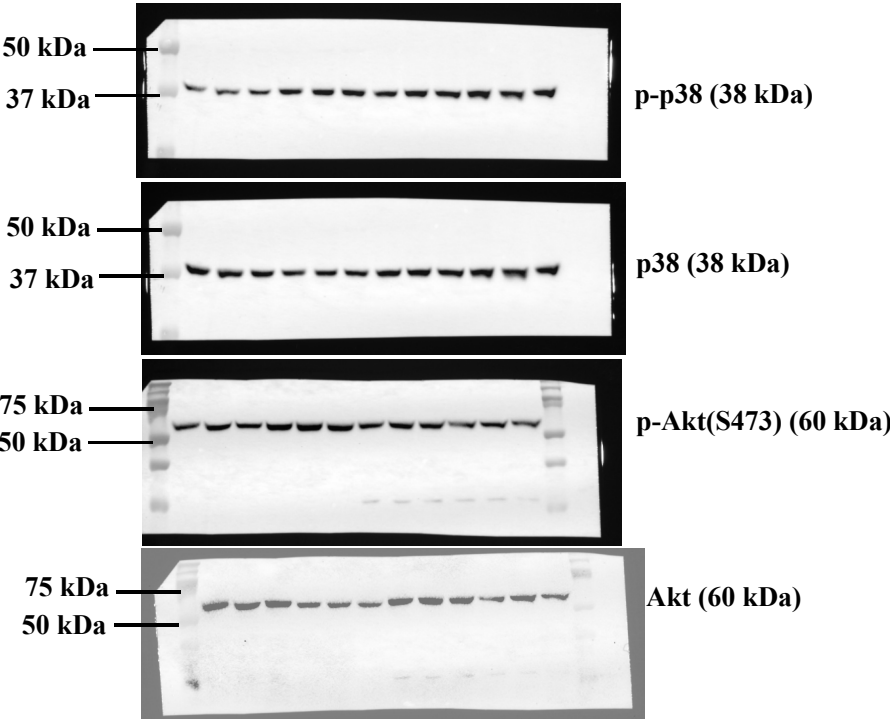

Supplement: Supplementary file 5 [file LSA-2023-02312_SdataFS6.pdf]

**FIGURE 6A.**

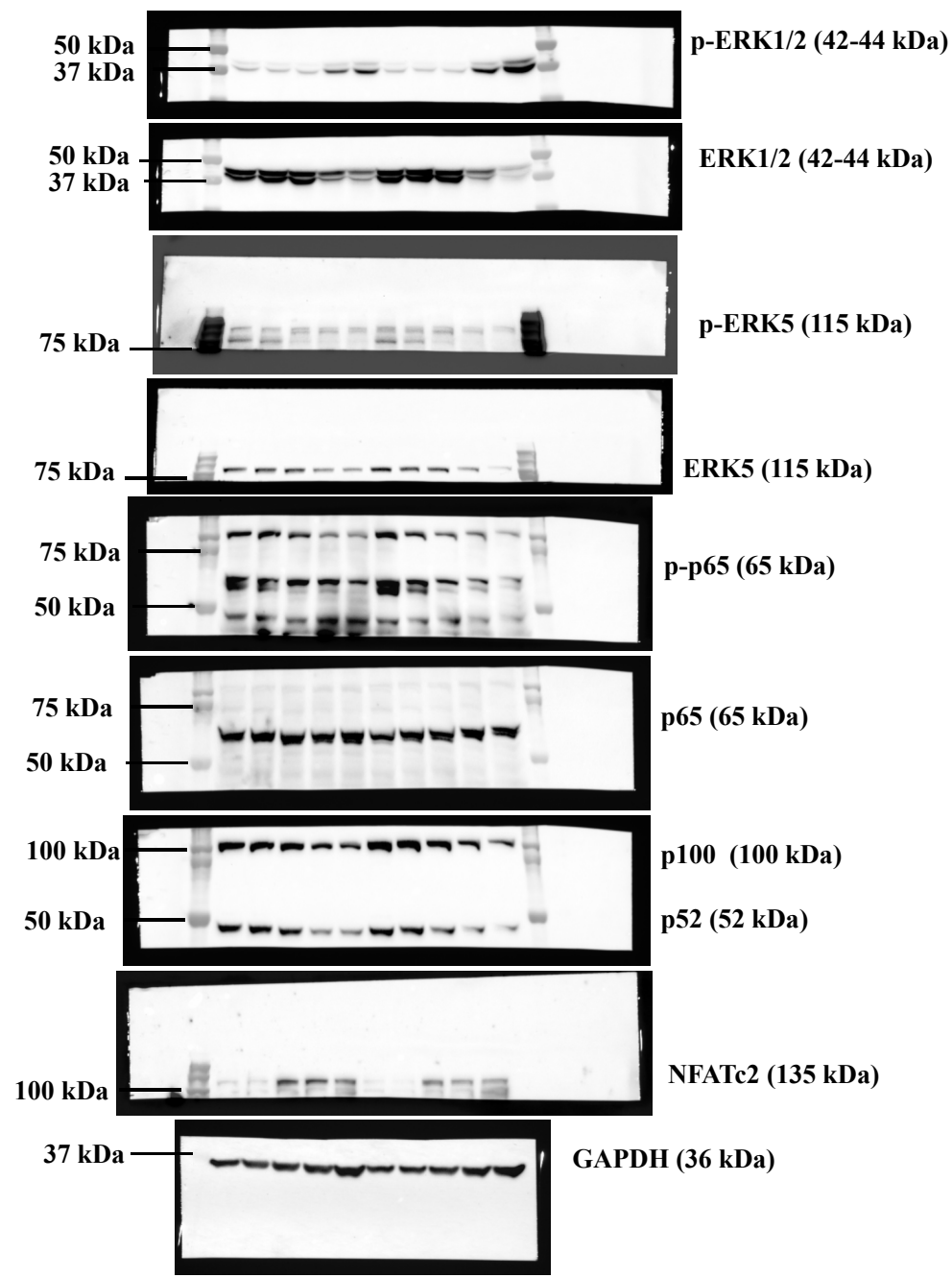

**FIGURE 6B.**

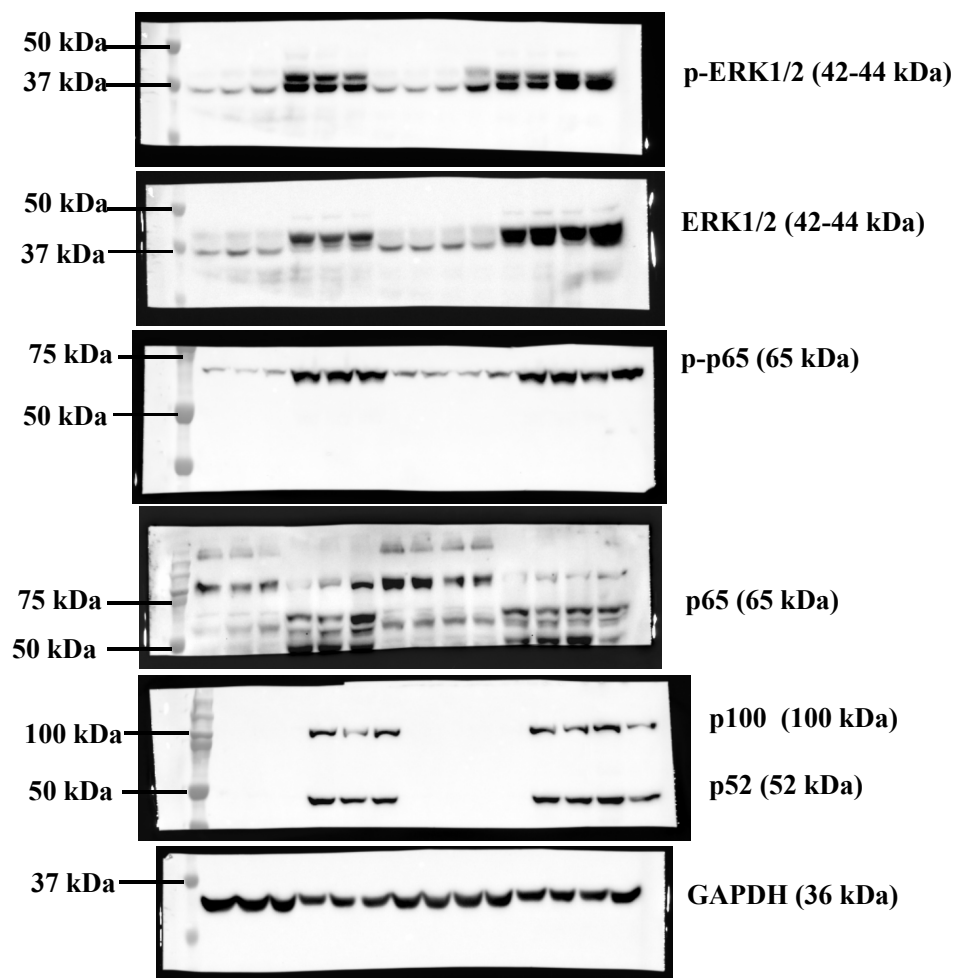

Supplement: Supplementary file 6 [file LSA-2023-02312_SdataF6.pdf]

**FIGURE 7**

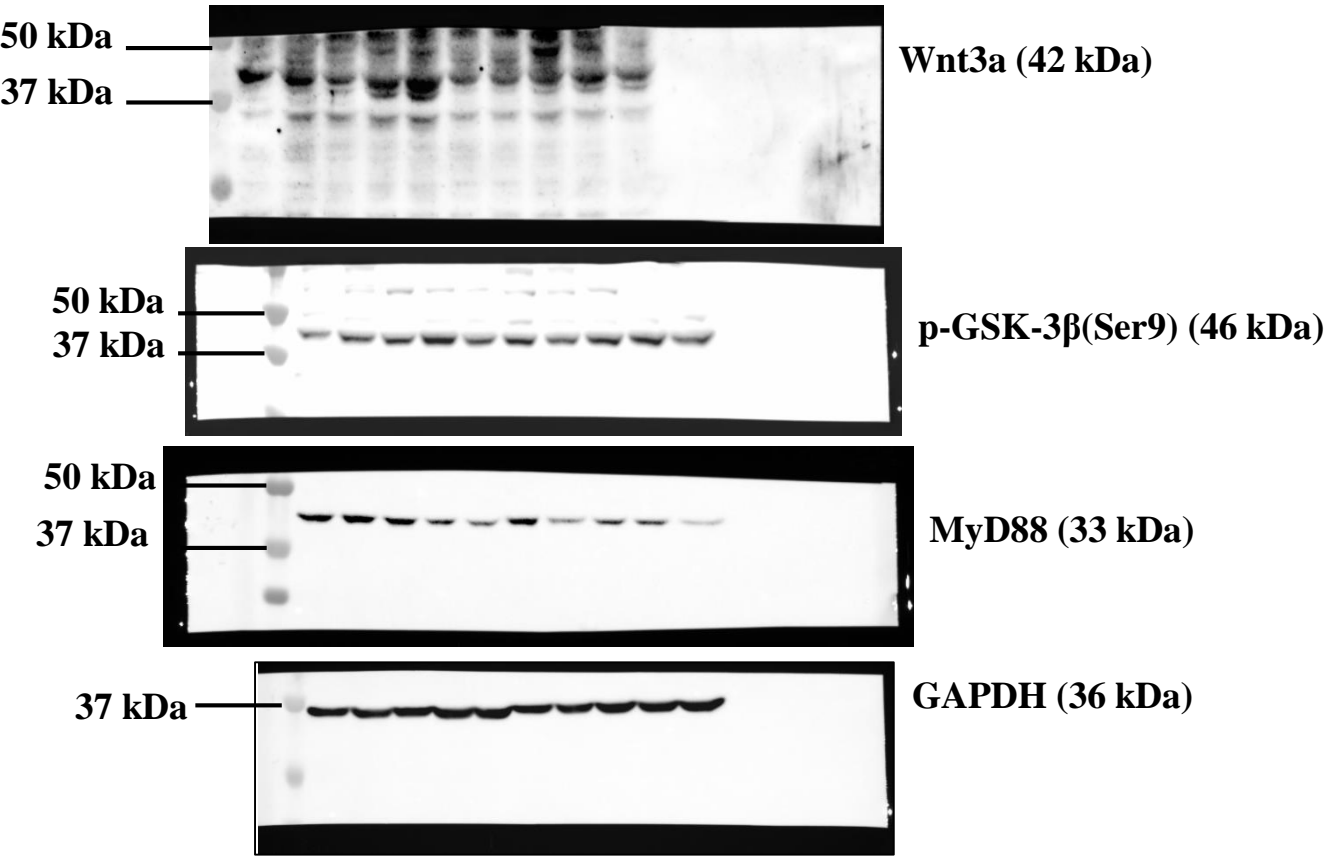

Supplement: Supplementary file 7 [file LSA-2023-02312_SdataF7.pdf]
