# Supplementary material for: Fn14 promotes myoblast fusion during regenerative myogenesis
Source: Life Sci Alliance. 2023 Oct 9;6(12):e202302312. doi: 10.26508/lsa.202302312 (PMC10561765; doi:10.26508/lsa.202302312)
Supplement: Supplementary file 8 [file LSA-2023-02312_TableS1.docx]

**Table S1.** Primers used for qRT-PCR experiments.

| **Gene Name** | **Forward primer (5'-3')** | **Reverse primer (5'-3')** |
| --- | --- | --- |
| Fn14 | AAGTGCATGGACTGCGCTTCTT | GGAAACTAGAAACCAGCGCCAA |
| Pax-7 | CAGTGTGCCATCTACCCATGCTTA | GGTGCTTGGTTCAAATTGAGCC |
| eMyHC | ACATCTCTATGCCACCTTCGCTAC | GGGTCTTGGTTTCGTTGGGTAT |
| MyoD | TGGGATATGGAGCTTCTATCGC | GGTGAGTCGAAACACGGATCAT |
| Myogenin | CAT CCA GTA CAT TGA GCG CCT A | GAG CAA ATG ATC TCC TGG GTT G |
| M-cadherin | TGGGCAGTCCCTGAGCCCAAA | TCCAGCGTGGCATTGAGGTACA |
| N-cadherin | CAGCAGATTTCAAGGTGGACGA | TCCTGGGTTTCTTTGTCTTGGG |
| BID-Integrin | CATCCCAATTGTAGCAGGCG | GAGACCAGCTTTACGTCCATAG |
| Caveolin-3 | GACCCCAAGAACATCAATGAGGAC | AGAAGGAGATACAGGCGAACAGGA |
| Myoferlin | CTACCAGAATGAGAATCGCTACCC | TACTCCCAGCCTTTCTCATCCA |
| ADAM12 | GGGCAAGAAGGCATAAGAGAGAGA | TGGTGAATGGGTCCTGGCTTAT |
| Myomaker | TATACTCCGGTCCCATAGGC | ATGCTCTTGTCGGGGTACAG |
| Myomerger-S | ACCAGCTTTCATGCCAGAAG | ATGTCTTGGGAGCTCAGTCG |
| Myomerger-L | CAGGAGGGCAAGAAGTTCAG | ATGTCTTGGGAGCTCAGTCG |
| Calpain-3 | TTTGTGAGAATCCCCGGTTTATC | CAGCCGCTCATTCAGGGTC |
| Calsequentrin-1 | CTGGCACTGCTGTTTGTACTG | GGGGGCTCATGGTAGAGGAG |
| Calsequentrin-2 | GACCGAGTGGTCAGCCTTTC | ACAGGTTCGTGGTAATAGAGACA |
| RyR1 | CAGTTTTTGCGGACGGATGAT | CACCGGCCTCCACAGTATTG |
| Calmodulin1 | TGGGAATGGTTACATCAGTGC | CGCCATCAATATCTGCTTCTCT |
| CamKIIb | CGTTTCACCGACGAGTACCAG | GCGTACAATGTTGGAATGCTTC |
| Wnt3 | TGGGCCTGTCTTGGACAAA | GCGATGGCATGCACGAA |
| Wnt4 | CTGGAGAAGTGTGGCTGTGA | GGACGTCCACAAAGGACTGT |
| Wnt5a | GGCATCAAGGAATGCCAGTA | GTACGTGAAGGCCGTCTCTC |
| Wnt7a | TGAAGAGGACCCAGTGACAGG | GGCGTACTGGTGTGTGTTGT |
| Wnt11 | GTAGGGCCTTCGCTGACAT | CGATGGTGTGACTGATGGTG |
| Fzd1 | GCCGGCTGAGCTTGGAACTT | AACCAAAGCAGCAGCAGCAGC |
| Fzd2 | CATCTCCATCCCGCTGTGCA | AGCACAGGAAGAAGCGCAGCTC |
| Fzd4 | GGCTACAACGTGACCAAGATGCC | GCACATTGGCACATAAACCGAAC |
| Fzd6 | GCGGCGTTTGCTTCGTT | CACAGAGGCAGAAGGACGAAGT |
| Axin-2 | TTTGGCACAGCTAGAGGAAG | TGGCTCTTTGTGATCTTCTGG |
| β-actin | CAGGCATTGCTGACAGGATG | TGCTGATCCACATCTGCTGG |
