# Supplementary material for: Fn14 promotes myoblast fusion during regenerative myogenesis
Source: Life Sci Alliance. 2023 Oct 9;6(12):e202302312. doi: 10.26508/lsa.202302312 (PMC10561765; doi:10.26508/lsa.202302312)
Supplement: Supplementary file 9 [file LSA-2023-02312_TableS2.docx]

**Table S2.** Antibodies used for various experiments.

| **Antibody** | **Source and Catalog no.** | **Dilution** | **Analysis** |
| --- | --- | --- | --- |
| Polyclonal rabbit-anti-TWEAK Receptor/Fn14 | Cell Signaling Technology # 44403 | 1:1000 | WB |
| Monoclonal rabbit-anti-GAPDH | Cell Signaling Technology # 2118 | 1:1000 | WB |
| Polyclonal rabbit-anti-phospho-p44/42 MAPK (Erk1/2) | Cell Signaling Technology # 9101 | 1:1000 | WB |
| Monoclonal rabbit-anti-p44/42 MAPK (Erk1/2) | Cell Signaling Technology # 4695 | 1:1000 | WB |
| Polyclonal rabbit-anti-Phospho-Erk5 | Cell Signaling Technology # 3371 | 1:1000 | WB |
| Polyclonal rabbit-anti-Erk5 | Cell Signaling Technology # 3372 | 1:1000 | WB |
| Monoclonal rabbit-anti-phospho-p65 NF-ĸB | Cell Signaling Technology # 3033 | 1:1000 | WB |
| Monoclonal rabbit-anti-total-p65 NF-ĸB | Cell Signaling Technology # 8242 | 1:1000 | WB |
| Polyclonal rabbit-anti-NF-ĸB p100/p52 | Cell Signaling Technology # 4882 | 1:1000 | WB |
| Monoclonal mouse-anti-NFATc2 | Santa Cruz Biotechnology # 7296 | 1:1000 | WB |
| Monoclonal rat IgG2A Clone # 217804.2R- anti- Wnt3a | R&D Systems # MAB13242 | 1:1000 | WB |
| Polyclonal rabbit-anti-phospho-Glycogen synthase kinase-3 (GSK-3) | Cell Signaling Technology # 9336 | 1:1000 | WB |
| Polyclonal goat-anti-MyD88 | R&D Systems, AF3109 | 1:1000 | WB |
| Polyclonalclonal rabbit-anti-phospho-p38 MAPK (Thr180/Tyr182) | Cell Signaling Technology #9211 | 1:1000 | WB |
| Polyclonal rabbit-anti-p38 MAPK | Cell Signaling Technology #9212 | 1:1000 | WB |
| Monoclonal rabbit-anti-phospho-Akt (Ser473) | Cell Signaling Technology #4060 | 1:1000 | WB |
| Polyclonal rabbit-anti-Akt | Cell Signaling Technology #9272 | 1:1000 | WB |
| Monoclonal mouse-anti-Pax7 | DSHB # PAX7 | 1:100 | WB/IF |
| Monoclonal mouse-anti-Myosin heavy chain (embryonic) | DSHB # F1.652 | 1:15 | WB/IF |
| Monoclonal mouse-anti-MyoD | Santa Cruz Biotechnology # 377460 | 1:200 | WB/IF |
| Monoclonal mouse-anti-Myogenin | DSHB # F5D | 1:100 | WB/IF |
| Monoclonal mouse-anti-Myosin heavy chain | DSHB # MF20 | 1:100 | WB/IF |
| Polyclonal rabbit-anti-Laminin | Sigma # L9393 | 1:500 | IF |
| Goat anti-Mouse IgG1 Alexa Fluor 568 | Life Technologies # A21124 | 1:1000 | IF |
| Goat anti-Mouse IgG2 Alexa Fluor 594 | Life Technologies # A211135 | 1:1000 | IF |
| Goat anti-Rabbit IgG Alexa Fluor 488 | Life Technologies # 11034 | 1:1000 | IF |
