# Supplementary material for: Fn14 promotes myoblast fusion during regenerative myogenesis
Source: Life Sci Alliance. 2023 Oct 9;6(12):e202302312. doi: 10.26508/lsa.202302312 (PMC10561765; doi:10.26508/lsa.202302312)
Supplement: Supplementary file 10 [file LSA-2023-02312_TableS3.docx]

**Table S3.** Mean TPM values for WT myoblasts incubated in growth medium.

| **Gene name** | **Mean TPM values** |
| --- | --- |
| Mymx | 166.1363 |
| Ripor2 | 2.677810301 |
| Il4ra | 32.59946177 |
| Il4 | 1.353711404 |
| Myog | 1319.010602 |
| Ehd1 | 62.6543135 |
| Cav3 | 108.0811797 |
| Mymk | 339.3554713 |
| Wnt9a | 23.36208778 |
| Wnt10b | 1.923614743 |
| Wnt4 | 1.585745791 |
| Wnt6 | 0.205362025 |
| Wnt2 | 0.147920737 |
| Wnt7b | 1.437116935 |
| Wnt5b | 2.336510562 |
